# Spontaneous multiple keloids: a case report

**DOI:** 10.11604/pamj.2024.48.110.43157

**Published:** 2024-07-17

**Authors:** Aishwarya Kishor Kedar, Vivek Dipakrao Alone, Rohini Amar Rathod, Shalaka Deepak Tupkari, Sree prada Bollineni, Anjana Ledwani, Ashwin Karnan, Harshith Beeravolu Reddy

**Affiliations:** 1 Datta Meghe Institute of Higher Education and Research, Sawangi Meghe, Wardha, Maharashtra, India,; 2 Shri Bhausaheb Hire Government Medical College and Hospital, Dhule, Maharashtra, India,; 3 Governement Medical College, Akola, Maharashtra, India

**Keywords:** Spontaneous, keloid, collagen bundles, triamcinolone, case report

## Abstract

An aberrant healing reaction to cutaneous injury or inflammation that spreads outside the original wound's boundaries causes keloidal scars. We present the case of a sixty-year-old male patient who initially came with complaints of respiratory system but had spontaneous lesions over his body for 50 years which remained undiagnosed. It was confirmed on histological examination to be keloids. The patient was treated with intralesional triamcinolone acetonide 40 mg plus an injection of lignocaine hydrochloride 2% in the ratio of 1:1 which provided him with a significant reduction in scar appearance over one month and thereby reducing his psychological burden. It is uncommon for spontaneous keloid scars to occur without any prior trauma or surgical intervention. Also, it details a manifestation of spontaneous keloid scars that manifest as numerous sizable lesions at various body locations. This study provides evidence in favor of such spontaneous appearance of keloid scars.

## Introduction

A keloid is a benign development of dense fibrous tissue that develops as a result of an aberrant healing response to a cutaneous injury and spreads beyond the original wound or inflammatory reaction [1]. Keloids are more likely to develop on body parts where the skin is strongly and/or repeatedly stretched, such as the anterior chest, shoulder, deltoid, mouth, and ears [2]. A keloid scar which is a clinical diagnosis requires determining a history of damage prior to the scar's development as a significant factor and spontaneous keloid scars, which grow without any previous trauma or surgical operation are considered rare [3].

We report a case of a sixty-year-old male patient who initially came with complaints of respiratory system but had lesions over his body for 50 years and denied any past history of trauma or surgery. Post histopathological examination the diagnosis of keloids was confirmed and the lesions were injected with triamcinolone acetonide 40 mg plus injection of lignocaine hydrochloride 2% in the ratio of 1:1 which provided him with a significant reduction in scar appearance over one month.

## Patient and observation

**Patient information:** a sixty-year-old male belonging to a rural area came to the outpatient department with the chief complaints of breathlessness and cough with mucoid expectoration for 15 days. The patient had a known case of chronic obstructive pulmonary disease for 5 years and was on a bronchodilator for the same. The patient denied any past history of trauma or surgery or any genetic disease. None of his family members reported to have any similar lesions on their body.

**Figure 1:**
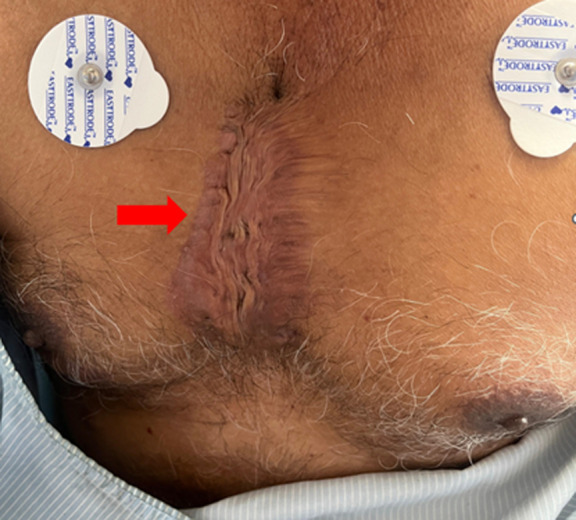
keloid on chest region: the lesion is single with well-defined margins, 8 cm x 6 cm in size hyperpigmented and raised (arrow)

**Figure 2:**
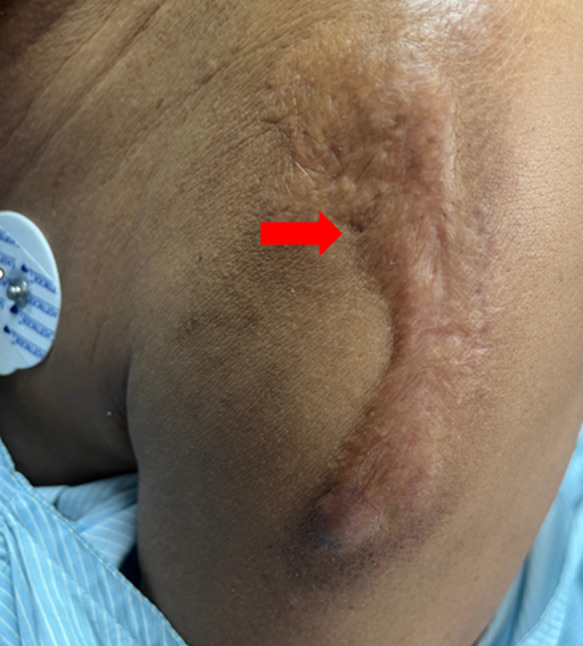
keloid on the left arm: the lesion is single with well-defined margins, 12 cm x 4 cm in size hyperpigmented and raised (arrow)

**Figure 3:**
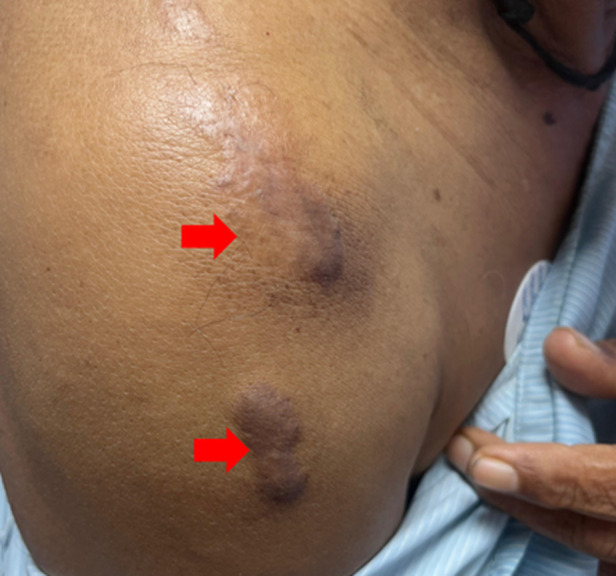
keloid on the right arm: the lesions are two in number with well-defined margins, 5 cm x 3.5 cm and 7 cm x 2 cm in size, hyperpigmented and raised (arrows)

**Figure 4:**
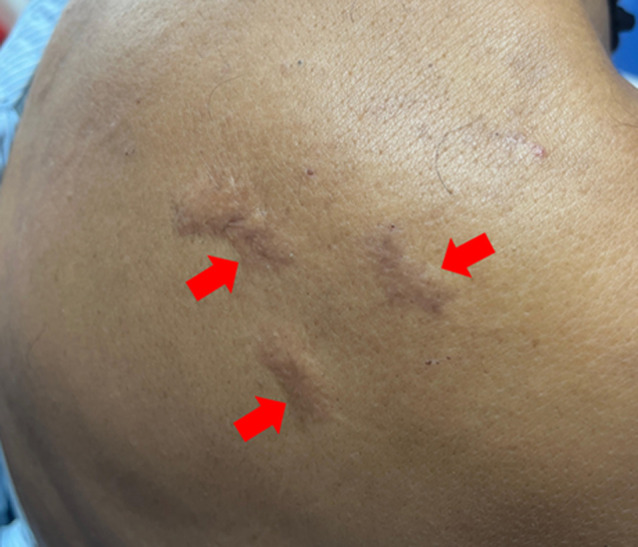
keloid on the back region: the lesions are three in number with well-defined margins, 3 cm x 1 cm, 2 cm x 1 cm, and 2.5 cm x 1 cm in size, hyperpigmented and raised (arrows)

**Clinical findings:** he was vitally stable and well-oriented to time, place, and person. On examination skin lesions were found on his chest (Figure 1), arms (Figure 2, Figure 3) and back (Figure 4). The skin lesions had been present for 50 years. These lesions first appeared on the chest followed by the arms and then on the back region.

They were painless, non-pruritic and gradually increased in size and number over time and had never gone into involution. The patient did not give any history of trauma to the sites of lesions or surgery. There was no history of similar complaints in his family. The lesions were well-defined, hyperpigmented, and skin-coloured plaques in asymmetric distribution and were of variable size and shape.

The other systems examined were unremarkable except for the presence of bilateral rhonchi on auscultation of the chest.

**Figure 5:**
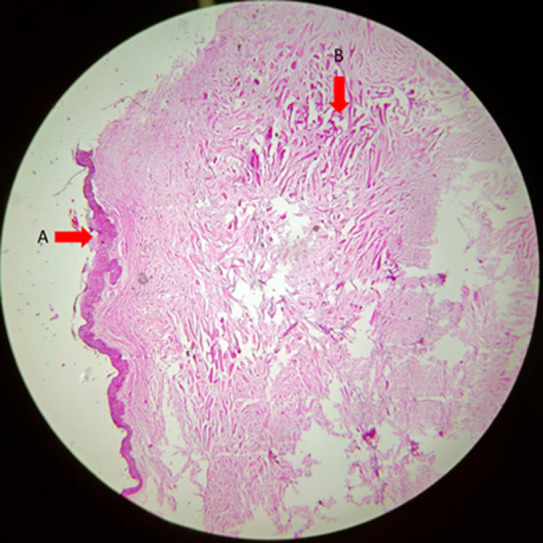
section studied shows stratified squamous lining: A) underneath shows a haphazard arrangement of long, broad closely packed collagen bundles; B) (hematoxylin and eosin *low power) (arrows)

**Figure 6:**
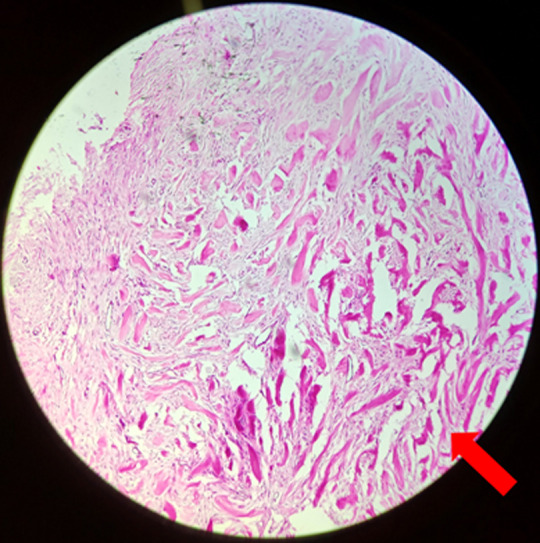
haphazard arrangement of long, broad closely packed collagen bundles (hematoxylin and eosin *high power) (arrow)

**Diagnostic assessment:** laboratory values were within normal limits. After providing symptomatic treatment to the patient, he was referred to the dermatology department where a biopsy was taken from the chest lesion and from the lesion on the left arm. Histopathological examination revealed haphazard arrangement of broad closely packed collagen bundles (Figure 5, Figure 6).

**Diagnosis:** from the appearance of lesion and histopathological examination a diagnosis of keloid was made.

**Therapeutic interventions:** the patient was treated with intralesional triamcinolone acetonide 40 mg plus injection lignocaine hydrochloride 2% in the ratio of 1:1. He was planned for monthly visits for the same therapy till regression. He was discharged from the hospital after symptomatic relief of his respiratory symptoms.

**Follow-up and outcome of interventions:** the patient had a significant reduction in the lesions when he followed up in the dermatology department after a period of 1 month.

**Patient perspective:** belonging to a rural setup, the patient had no access to proper diagnosis and treatment before coming to our hospital through medical camps. His respiratory complaint brought him to the hospital which led to identifying the lesions on his body which remained undiagnosed for 50 years. The patient had a psychological load as a result of the lesions for so many years which were unsightly and was happy to get it diagnosed and treated.

**Informed consent:** written informed consent was taken before initiating the case report.

## Discussion

Histologically, keloids are pathological scars that are characterised by an excessive aggregation of collagen type I and fibroblasts within the inflammatory reticular dermis. Their erythematous and pruritic leading edge allows them to continuously expand and invade the surrounding healthy skin beyond the initial wound boundary, among other clinical features. Although keloids are widespread worldwide, not enough research has been done on their epidemiology. Regional and ethnic differences may have an impact on keloid rates. Significant regional variations in the keloid rate could be a reflection of racial variations in skin colour. First, according to Louw, Asians and Blacks are more prone than Caucasians to develop keloid development. Furthermore, according to two studies, Black people are significantly more likely to develop Acne Keloidalis Nuchae (AKN) than Asians, a particular type of keloid [4]. Keloids typically develop on extremely tense, mobile settings [4,5].

Analysis of 1,500 keloids in 483 Japanese patients revealed that they tended to form on the anterior chest region (48.9%), scapular regions (26.9%), lower jaw/neck region (12.1%), upper arm (4.8%), dorsal regions (2.5%), lower abdomen (1.9%), femoral regions (1.7%), knee (0.5%), and upper abdomen (0.5%), keloids created by artificial wounds, i.e. those created by surgery and piercing, were excluded. The fact that the keloids in some areas grow into particular forms indicates that skin tension plays a role in the formation of keloids. As a result, the symmetrical butterfly forms formed by anterior chest keloids represent the main stretching orientations of the chest skin brought on by upper arm movements [5]. There is proof of additive, oligogenic, or autosomal dominant inheritance in families from earlier genetic research. Gene expression investigations in keloid cells and families have produced highly diverse results, which further suggests that keloids are caused by a heterogeneous genetic event. One gene linked to keloid risk is ASAH1 [6]. Rubinstein-Taybi syndrome (RSTS), Ehlers-Danlos syndrome, Lowe syndrome, new X-linked syndrome, Dubowitz syndrome, Noonan syndrome, and Goeminne syndrome are among the illnesses connected to a higher incidence of keloid. It is evident that both internal genetic and exterior environmental variables contribute to the development of keloids, regardless of whether the lesions develop spontaneously or as a result of trauma [4].

Clinical examination is usually sufficient to definitively identify keloids. The primary indicators for diagnosis are morphology, topography, and history. Rarely, unusual or dubious clinical patterns call for a skin biopsy for histological confirmation. Histopathological investigations show that keloid keratinocytes express more epithelial-mesenchymal transition (EMT) markers. Fibroblasts are found in greater quantities in the keloid dermis, according to histopathological investigations. Other types of dermal cells found in keloids are fibrocytes and myofibroblasts, both of which are more numerous [7]. A tool developed by the Japan Scar Workshop (JSW) can be used to objectively diagnose hypertrophic scars and keloids. The JSW Scar Scale (JSS) is a technique used to score the risk factors of individual patients as well as the affected areas. A lesion with a score of 16 points or higher on the Japan Scar Workshop (JSW) Scar Scale is more likely to be a keloidal tumour. Collagen fibres that are dense and consistently pigmented in keloids are referred to as hyalinized or keloidal collagen. These dermal nodules, are combined with keloidal collagen. When keloids are diagnosed by ultrasonic elastography, they appear as tougher regions compared to the surrounding tissues. Moreover, ultrasound imaging is useful for assessing how a treatment is affecting the keloid scar. This imaging modality depicts keloids as low echo areas compared to the surrounding dermis. The inside of the lesion is often heterogeneous [8].

Intralesional or topical medications that function at the cellular level have replaced crude, intrusive techniques like radiation and gross excision in the management of hypertrophic scars and keloids. Surgical excision, laser, intralesional injections, silicone and pressure dressings, topical mitomycin C, oral medications, extracorporeal shockwave therapy, autologous fat grafting, photodynamic therapy, radiation, RNA-based therapies, stem cell therapy, and cryotherapy are among the available treatment options at the moment [9,10]. Surgical excision was one of the first methods used to treat hypertrophic scars and keloids. The extremely high recurrence rate of surgery as a stand-alone treatment is its main drawback. For keloid-prone patients, surgery alone results in epidermal harm even when the majority of the scar may be excised. The argon laser was initially employed in keloid therapy; however, due to its limited ability to flatten scars, it was replaced by carbon dioxide, Nd: YAG, and flashlamp-pumped pulsed-dye lasers (PDLs). Because of the ensuing hypopigmentation and postoperative discomfort, patients may find other treatment modalities more appealing than cryotherapy [10].

Corticosteroid injections have long been used to treat and prevent hypertrophic scars and keloids. Triamcinolone acetonide injections limit fibroblast proliferation while promoting collagen degradation. One study found that intralesional injection of triamcinolone acetonide resulted in clinical improvement in 72% of patients and complete flattening in 64% of lesions. Adverse effects of intralesional corticosteroids include hypopigmentation, cutaneous atrophy, telangiectasia, and injection site discomfort [10].

Pressure or occlusive dressings have been used as both a substitute and a supplement to surgical excision. The mechanism of action is uncertain, but it has been proposed that pressure and scar hydration cause fibroblast alteration and collagen degradation. Interferon therapy has the potential to be beneficial in the treatment of aberrant scars because it reduces the synthesis of collagen types I and III. Multiple studies employing fluorouracil as monotherapy revealed that more than three-quarters of patients achieved a scar size decrease of at least 50%. Bleomycin therapy significantly improved scar height and pliability while also reducing erythema, pruritus, and discomfort [10]. Botulinum toxin A can reduce scar proliferation by lowering muscle tension during wound healing, interrupting fibroblast cell cycles in the non-proliferative period, and modifying TGF-β1 expression [9].

Although there are numerous therapeutic options for keloids, they continue to provide a clinical challenge for both patients and clinicians. Given the intricate process of keloid formation, a fuller knowledge of the molecular pathways that drive keloid development and recurrence might open more opportunities for designing new treatments [9].

## Conclusion

The presented case explains the diagnostic complexity of keloids. As our patient denied any previous history of trauma or procedures over the affected area, this case report supports the fact that keloid scars can appear spontaneously. Through histopathological examination, a diagnosis of keloid scars was made, emphasizing its crucial role. In our case, the patient was treated with intralesional triamcinolone acetonide 40 mg plus an injection of lignocaine hydrochloride 2% in the ratio of 1: 1 which is the most frequently used modality and also provided beneficial results to the patient.
